# *IFT46* gene promoter-driven ciliopathy disease model in zebrafish

**DOI:** 10.3389/fcell.2023.1200599

**Published:** 2023-06-08

**Authors:** Mi-Sun Lee, Hye-Jeong Han, Tae-Ik Choi, Kang-Han Lee, Amartuvshin Baasankhuu, Hyun-Taek Kim, Cheol-Hee Kim

**Affiliations:** ^1^ Department of Biology, Chungnam National University, Daejeon, Republic of Korea; ^2^ Michigan Neuroscience Institute (MNI), University of Michigan, Ann Arbor, MI, United States; ^3^ Soonchunhyang Institute of Medi-Bio Science (SIMS), Soonchunhyang University, Cheonan-Si, Republic of Korea; ^4^ Department of Integrated Biomedical Science, Soonchunhyang University, Cheonan-Si, Republic of Korea

**Keywords:** *IFT46*, ciliopathy, GAL4/UAS system, NTR/MTZ system, zebrafish

## Abstract

Ciliopathies are human genetic disorders caused by abnormal formation and dysfunction of cellular cilia. Cilia are microtubule-based organelles that project into the extracellular space and transduce molecular and chemical signals from the extracellular environment or neighboring cells. Intraflagellar transport (IFT) proteins are required for the assembly and maintenance of cilia by transporting proteins along the axoneme which consists of complexes A and B. IFT46, a core IFT-B protein complex, is required for cilium formation and maintenance during vertebrate embryonic development. Here, we introduce transgenic zebrafish lines under the control of ciliated cell-specific *IFT46* promoter to recapitulate human ciliopathy-like phenotypes. We generated a *Tg(IFT46:GAL4-VP16)* line to temporo-spatially control the expression of effectors including fluorescent reporters or nitroreductase based on the GAL4/UAS system, which expresses GAL4-VP16 chimeric transcription factors in most ciliated tissues during embryonic development. To analyze the function of *IFT46*-expressing ciliated cells during zebrafish development, we generated the *Tg(IFT46:GAL4-VP16;UAS;nfsb-mCherry)* line, a ciliated cell-specific injury model induced by nitroreductase (NTR)/metrodinazole (MTZ). Conditionally, controlled ablation of ciliated cells in transgenic animals exhibited ciliopathy-like phenotypes including cystic kidneys and pericardial and periorbital edema. Altogether, we established a zebrafish NTR/MTZ-mediated ciliated cell injury model that recapitulates ciliopathy-like phenotypes and may be a vertebrate animal model to further investigate the etiology and therapeutic approaches to human ciliopathies.

## 1 Introduction

Cilia are hair-like, highly conserved microtubule-based organelles which extend from the surface of most cell types of the human body and exist in a diverse range of organisms from protozoa to primates (Fliegauf et al., 2007). In vertebrates, cilia are present in most cell types and perform diverse biological functions (Ishikawa and Marshall, 2011). Cilia are classified as either motile or non-motile (primary cilia); motile cilia generate force for sperm cell motility and fluid flow over the surface of epithelial cells, while non-motile primary cilia act as an antenna for sensing extracellular signals (Eggenschwiler and Anderson, 2007; Gerdes et al., 2009; Veland et al., 2009; Goetz and Anderson, 2010).

Ciliary dysfunction is the cause of an increasing number of single-organ diseases and complex syndromic forms including hydrocephalus; infertility; airway diseases; polycystic diseases of the kidney, liver, and pancreas; and retinal diseases and defects in hearing and smell (Fliegauf et al., 2007). Cilium loss and/or abnormal cilium development or function lead to a group of human genetic disorders collectively called ciliopathies, which include polycystic kidney disease (PKD), nephronophthisis, retinitis pigmentosa, polydactyly, primary ciliary dyskinesia (PCD), and developmental delays such as Bardet–Biedl syndrome, Joubert syndrome, and Meckel syndrome (Hildebrandt et al., 2011; Falk et al., 2015). Clinical manifestations of ciliopathies can increase in most tissue types during development and throughout life (Badano et al., 2006; McIntyre et al., 2013; Reiter and Leroux, 2017; McConnachie et al., 2021); however, the genetic and biological cellular controls of ciliogenesis remain poorly understood.

Genes causing ciliopathies are highly conserved and involved in the maintenance of cilia. Their encoded proteins interact dynamically with multiple protein complexes that are expressed in the cilia, basal body, centrosome, and mitotic spindle in a cell cycle-dependent manner (Scholey and Anderson, 2006). The assembly, maintenance, and function of cilia require bidirectional movement of protein complexes along microtubule-based axonemes. This motor system is named intraflagellar transport (IFT) (Rosenbaum and Witman, 2002). Due to the inability of cilia to synthesize their own proteins, IFT uses IFT transporters as a train for vesicular trafficking of these proteins throughout the cilia (Taschner and Lorentzen, 2016). IFT is a conserved process in eukaryotes, which assembles, maintains, disassembles, and transduces cilium-generated signaling. IFT is composed by the IFT-A and IFT-B protein complexes. The motor-based trains of the IFT complex move along the microtubule axoneme to perform anterograde transport (base to tip) by the IFT-B complex and kinesin-2 motors, while the IFT-A complex with dynein motors is responsible for retrograde transport (tip to base) of ciliary proteins (Rosenbaum and Witman, 2002). The IFT system regulates protein entry and exit, which is required for ciliary maintenance and growth and for populating the organelle with functional receptors and other sensory proteins. Different IFT proteins play various roles in the IFT complexes. However, many reports discovered that the functions of IFT proteins are not only limited to cilia. IFT20 plays a role in the mammalian immune synapse (Vivar et al., 2016; Galgano et al., 2017), and IFT88 plays an independent role during cell migration (Boehlke et al., 2015).

The IFT-B complex consists of nine core components and six peripheral subunits (Taschner et al., 2016). Mutations in genes encoding IFT components lead to ciliary defects and have implicated numerous ciliopathies in many tissues (Ishikawa and Marshall, 2017). IFT46 is a core component of the IFT-B protein complex and is required for the formation of all cilia. IFT46 mutants of *C. reinhardtii* and *C. elegans* are incapable of assembling cilia, demonstrating that IFT46 plays an essential role in ciliogenesis (Hou et al., 2007). IFT46 forms a stable trimetric sub-complex within the IFT-B core complex together with IFT52 and IFT88 (Lucker et al., 2010). IFT46 is expressed in many ciliated tissues such as pronephric ducts, eyes, otic vesicle, spinal canal, nose, and sensory hair cells during zebrafish embryonic development. Knockdown of IFT46 by morpholino caused the formation of fewer and shorter cilia in zebrafish and *Xenopus*. In addition, IFT46 knockout mice exhibited defects in left–right axis patterning and short cilia (Lee et al., 2015; Park et al., 2016). Another study in *Paramecium* shows IFT46 plays an important role in trafficking IFT proteins between cilia and cytoplasm (Shi et al., 2018). These previous studies suggest that IFT46 plays an essential role in cilium development.

Targeted cell ablation is a powerful technique to study tissue regeneration and examine how specific cell lineages contribute to development and disease *in vivo* (Gregoire and Kmita, 2014). In zebrafish, NTR-mediated cell ablation has proven useful. Zebrafish-expressing NTR in specific cell compartment sensitizes these cells to MTZ-mediated cell ablation which can be used for studying tissue regeneration and investigating cell function (Curado et al., 2007; Pisharath et al., 2007; White and Mumm, 2013; Gregoire and Kmita, 2014). A bacterial NTR enzyme converts non-toxic prodrug MTZ into a DNA damaging cytotoxin, which causes apoptotic cell death of the NTR-expressing cells. The generation of transgenic lines to control NTR expression has facilitated targeted ablation of various cell types such as hepatocytes, podocytes, and neutrophils (Curado et al., 2008; Zhou and Hildebrandt, 2012; Huang et al., 2013; Hall et al., 2022).

In this study, we introduce a transgenic zebrafish to generate ciliopathy phenotypes by driving an NTR enzyme under the control of an IFT gene promoter. We isolated and characterized the 2.4 kb upstream regulatory sequence of *IFT46*, which is sufficient to drive endogenous *IFT46* expression in various ciliated tissues. Moreover, we generated a ciliated-cell-specific injury model, *Tg(IFT46:GAL4-VP16;UAS;nfsb-mcherry)* line, which is mediated by the NTR/MTZ system. Our findings showed that the MTZ-treated transgenic embryos exhibited ciliopathy-like phenotypes such as cystic kidneys and pericardiac and periorbital edema, which are shown in *IFT46* CRISPRant.

## 2 Materials and methods

### 2.1 Zebrafish maintenances

Zebrafish were maintained at 28.5°C with a 14/10 h light and dark cycle. Wild-type embryos were cultured in egg water (40 g of sea salts added to 1 L distilled water and 60 μg/mL final concentration) and treated with PTU (1-phenyl-2-thiourea) (Sigma-Aldrich, St. Louis, MO, United States) to suppress pigmentation at the bud stage. Zebrafish were obtained from the Zebrafish Center for Disease Modeling (ZCDM; Daejeon, Republic of Korea). All experimental protocols and procedures were approved and conducted according to the approved guidelines and regulations of the Animal Ethics Committee of Chungnam National University (approval number: CNU 00191).

### 2.2 Isolation of the *IFT46* promoter and plasmid construction

To find the enhancer elements of the zebrafish *IFT46* (GenBank Acc. No.: NM_001346250) gene, we used the TFSEARCH (searching transcription factor-binding sites, ver 1.3) program (http://diyhpl.us/∼bryan/irc/protocol-online/protocol-cache/TFSEARCH.html). The wild-type zebrafish genomic DNA was used as a template for PCR to isolate *IFT46* promoter regions. We used the following primer set: 5′-CCG​TCG​ACT​CTA​CAG​AGC​TTG​GCA​CGA​ATG​TC-3′ (forward primer) and 5′-ACG​GAT​CCT​GTC​GCT​CGG​ACC​TCT​CCA​T-3′ (reverse primer). The PCR fragment was ligated into pCS2 + EGFP and pCS2 + mRFP vectors followed by the ligation of the 2.4 kb 5′ upstream regions of the *IFT46* gene. To generate the *Tg(IFT46:GAL4-VP16)* transgenic line, 2.4 kb *IFT46* 5′ upstream fragments were ligated into the *mini-pTol2:GAL4-VP16* vector.

### 2.3 Microinjection of plasmids into the zebrafish embryos

Zebrafish embryos were injected with purified mini-Tol2*-IFT46:GAL4-VP16* constructs (50 ng/μL) with transposase mRNA (200 ng/μL) at the one-cell stage. To generate stable transgenic lines, injected embryos increased to adulthood and out-crossed with wild-type zebrafish. The *Tg(IFT46:GAL4-VP16)* fish were crossed with *Tg(UAS:EGFP)*, *Tg(UAS:mGFP;cmlc2:GFP)*, and *Tg(UAS:nfsb-mCherry)* lines. The embryos were screened based on GFP and/or mCherry reporter expression. In addition, the *IFT46:GAL4-VP16* driver line was identified by genomic DNA PCR using the following primer set: 5′-GAG​GAT​CCG​CCA​CCA​TGA​AGC​TAC​TG-3′ (forward primer) and 5′-TCT​AGA​AGC​TAC​CCA​CCG​TAC​TCG​TCA​AT-3′ (reverse primer).

### 2.4 Whole-mount two-color *in situ* hybridization

To synthesize anti-sense RNA probe for whole-mount *in situ* hybridization, cDNA was PCR-amplified from total RNA extracted at 24 hpf embryos. The PCR fragments were cloned into the pGEM®-T Easy Vector (Promega, Madison, WI, United States). The cloned vectors were linearized with the *NcoI* restriction enzyme and then transcribed *in vitro* using SP6 RNA polymerase (Thermo Scientific, Waltham, MA, United States) with digoxigenin-labeled UTP (Roche, Basel, Switzerland). The following primers were used: *odf3b* (NCBI, acc. no. NM_199958) forward 5′-AGA​CGT​CAT​GTC​ACC​TGT​G-3′ and *odf3b* reverse 5′-ATG​ACC​TGC​TTA​ATC​TTC​ACC​ATC​C-3′. Whole-mount *in situ* hybridization was performed as described previously (Thisse and Thisse, 2008).

### 2.5 Whole-mount immunostaining and cryosection

For whole-mount immunofluorescence staining, embryos were fixed in 4% paraformaldehyde at 4°C overnight (Kimmel et al., 1995). After three times washing with PBS, embryos were dehydrated with 100% methanol. The embryos were permeabilized in acetone for 7 min at −20°C, followed by three washes with PBST. After blocking for 30 min in 5% horse serum in PBST, embryos were incubated with the anti-acetylated α-tubulin antibody (1/1,000) (Sigma-Aldrich, St. Louis, MO, United States) at 4°C overnight. On the next day, zebrafish embryos were incubated with the Alexa Fluor 488-conjugated secondary antibody (Invitrogen, Carlsbad, CA, United States). To stain nuclei, the embryos were incubated for 10 min with DAPI (Sigma-Aldrich, St. Louis, MO, United States) and washed in PBS. Images were captured using Leica microscope. For cryosection, embryos were fixed in 4% paraformaldehyde at 4°C overnight, washed in PBS, and then infiltrated with 10%, 20%, and subsequently 30% sucrose/PBS solution at 4°C overnight. Embryos were placed in the OCT compound (Tissue-Tek, Sakura Finetek, United States) and solidified in a plastic mold on dry ice. Sections (10 µm thickness) were obtained using a cryostat.

### 2.6 Acridine orange staining

For the detection of apoptotic cells in living embryos, embryos were incubated in 1 μg/mL acridine orange (Sigma-Aldrich, St. Louis, MO, United States) in egg water for 30 min in dark condition and then washed with fish water thrice.

### 2.7 Cell ablation using MTZ treatment

Metronidazole (Sigma-Aldrich, St. Louis, MO, United States) was dissolved in 0.1% DMSO in egg water. For ciliated cell-specific ablation, *Tg(IFT46:GAL4-VP16;UAS:nfsb-mCherry)* embryos were incubated in 5–10 mM final concentration of MTZ solution at 28.5°C for 12–72 h at different stages and changed fresh solution every 12 h. All these treatments were placed in the dark area for the light sensitivity of MTZ. At indicated times, MTZ solution was removed and replaced with egg water for recovery.

### 2.8 Generation of *IFT46* F0 CRISPRants

Gene editing was performed as previously described (Vejnar et al., 2016). CHOPCHOP (http://chopchop.cbu.uib.no/) was used to design gRNAs targeting exons 7–9 of the *IFT46* gene. The gRNAs were transcribed using purified PCR products as a template, and the MEGAscript T7 Transcription Kit (Thermo Fisher Scientific #AM1334) was used for *in vitro* transcription. Primers for making *IFT46* F0 CRISPRants are as follows: T7 universal gRNA primer: 5′-AAA​AGC​ACC​GAC​TCG​GTG​CCA​CTT​TTT​CAA​GTT​GAT​AAC​GGA​CTA​GCC​TTA​TTT​TAA​CTT​GCT​ATT​TCT​AGC​TCT​AAA​AC-3′; *IFT46*-sg#ex7 primer: 5′-TAA​TAC​GAC​TCA​CTA​TAG​GCG​GAA​AGC​CTG​ACA​ATC​TGT​TTT​AGA​GCT​AGA​A-3′; *IFT46*-sg#ex8 primer: 5′-TAA​TAC​GAC​TCA​CTA​TAG​GGA​TGC​ACT​TCT​CTT​TCA​CGT​TTT​AGA​GCT​AGA​A-3′; and *IFT46*-sg#ex9 primer: 5′-TAA​TAC​GAC​TCA​CTA​TAG​GGC​TGT​CAA​TGT​CTG​GCA​TGT​TTT​AGA​GCT​AGA​A-3′. The Cas9 mRNA was *in vitro* transcribed using the Not1-digested *pCS2+Cas9* vector. The gRNAs and Cas9 mRNA were co-injected into one-cell stage embryos. Genomic DNA was extracted from the *IFT46* gRNAs and Cas9-injected embryos. The targeted region was amplified from genomic DNA using the following primers: *IFT46* forward 5′-TCT​CAC​ACT​TGA​AGC​ACT​GC-3′ and *IFT46* reverse 5′- GAG​AAC​CAA​CCT​TCC​CCA​GA-3′. The PCR products were purified, and T7 endonucelase I (T7E1) assays were performed as previously described (Sung et al., 2014).

### 2.9 Statistical analyses

Statistical analyses were performed using GraphPad Prism software (GraphPad, San Diego, DA, United States). The non-parametric two-tailed Mann–Whitney test was used for comparison. Statistical significance was set at *p* < 0.05. Error bars are the SD.

## 3 Results

### 3.1 Isolation and characterization of the ciliated cell-specific *IFT46* promoter

**FIGURE 1:**
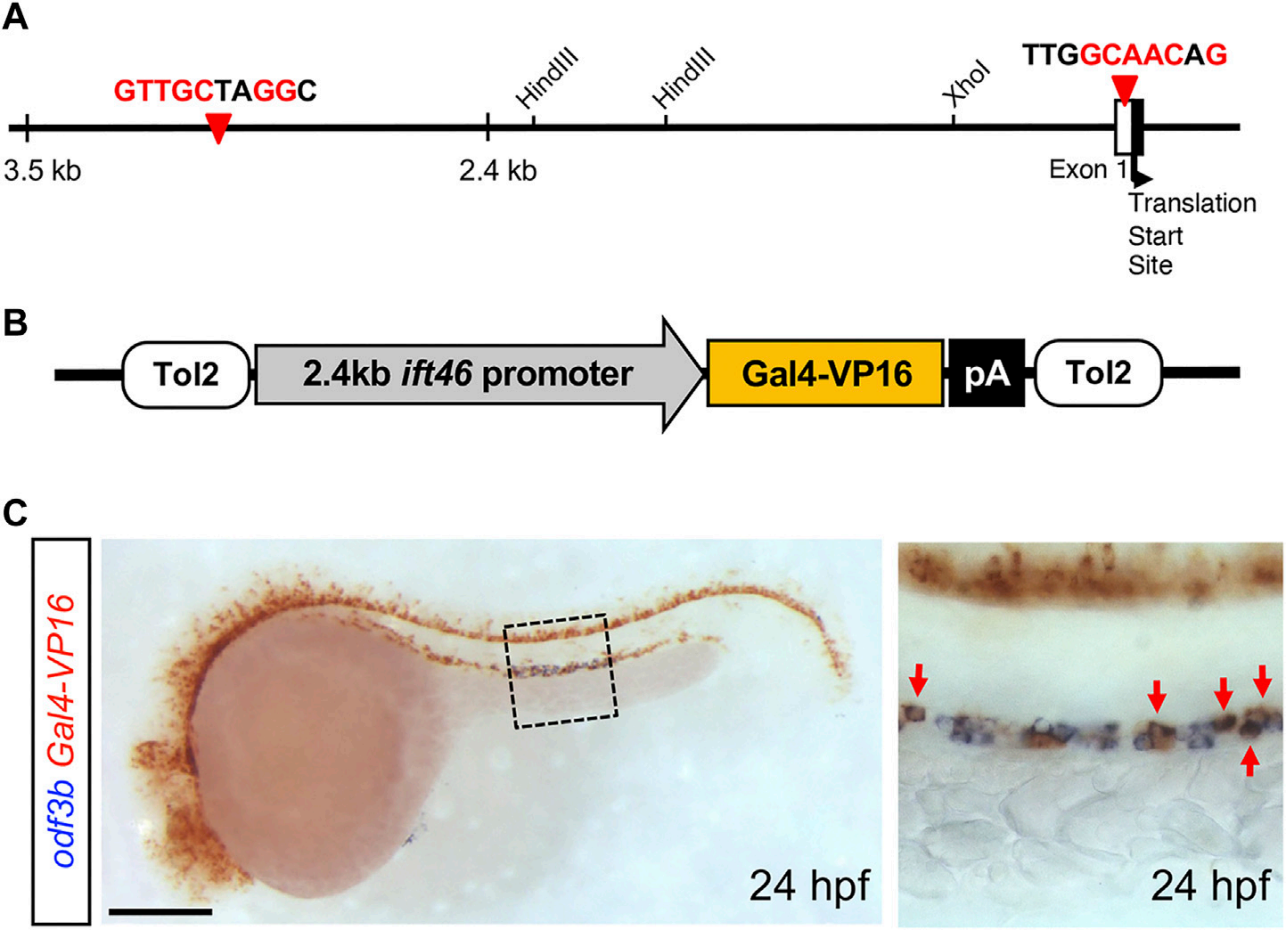
Transient expression of *IFT46:GAL4-VP16* in ciliated cells. **(A)** Schematic representation of the 5′ upstream regulatory region of the *IFT46* gene that contains putative two *RFX2* transcription factor-binding site sequences (red arrowheads). **(B)** Schematic view of the Tol2-based construct containing GAL4-VP16 under the control of the 2.4 kb *IFT46* promoter. **(C)** Two-color *in situ* hybridization of the *GAL4-VP16* chimeric transcription factor (red) with *odf3b* (blue) at 24 hpf. *GAL4-VP16* transcripts are expressed in multi-ciliated cells (box region) in the distal segment of the pronephric duct (red arrows). Scale bar: 200 μm **(C)**.

We have previously reported that the *IFT46* gene is expressed in ciliary organs and is involved in cilium development in vertebrates such as zebrafish and mice (Lee et al., 2015). To generate stable inducible transgenic lines under the control of the *IFT46* promoter, we first checked upstream regulatory sequences from a translation start-site of *IFT46* and found two putative *RFX2* transcription factor-binding sites (Figure 1A). The regulatory factor X (RFX) family of the transcription factors is known to play a crucial role in ciliogenesis. RFX2 is a member of the winged helix transcription factor, which is expressed in motile cilia in mice and zebrafish. *RFX2* deficiency in zebrafish leads to defects in LR patterning (Bisgrove et al., 2012). Thus, we isolated the 2.4 kb upstream region of the *IFT46* promoter*,* which included two *RFX2* transcription factor-binding sites. To examine whether regulatory elements can recapitulate the endogenous expression of the *IFT46* gene, we microinjected *IFT46:GFP* and *IFT46:mRFP* plasmids into embryos (Supplementary Figure S1A). GFP and mRFP reporter expression was restricted to the ear, photoreceptor cell layer in the eye, pronephric duct, spinal canal, and olfactory organs in the central nervous system (CNS) where cilia are present during embryogenesis (Supplementary Figures S1B, C). These results indicate that the 2.4 kb upstream sequence of *IFT46* contained regulatory elements sufficient to drive *IFT46* expression in ciliated cells during embryonic development in zebrafish.

### 3.2 Establishment of the Tg(*IFT46:GAL4-VP16*) zebrafish line

The GAL4/UAS system is a powerful tool to modulate gene expression in a cell-specific and temporally restricted manner (Asakawa et al., 2008; Halpern et al., 2008). To establish *IFT46* promoter-derived GAL4 transgenic zebrafish, we injected a construct, consisting of the GAL4-VP16 heteromeric transcription factor under the control of the *IFT46* promoter, to activate the reporter and toxin genes in a ciliated cell-specific manner (Figure 1B). To find stable transgenic lines, we examined PCR using GAL4 gene-specific primers and obtained 700 bp size of PCR amplicon (Supplementary Figure S2A). To confirm whether GAL4/VP16 expression can recapitulate *IFT46* expression, we examined GAL4 mRNA expression by whole-mount *in situ* hybridization. The GAL4 transcripts were detected in ciliary organs where *IFT46* was expressed during early development (Supplementary Figures S1B–F) (Lee et al., 2015), indicating that *Tg(IFT46:GAL4-VP16)* can be used as a driver line for ciliary organ-specific expression.

**FIGURE 2:**
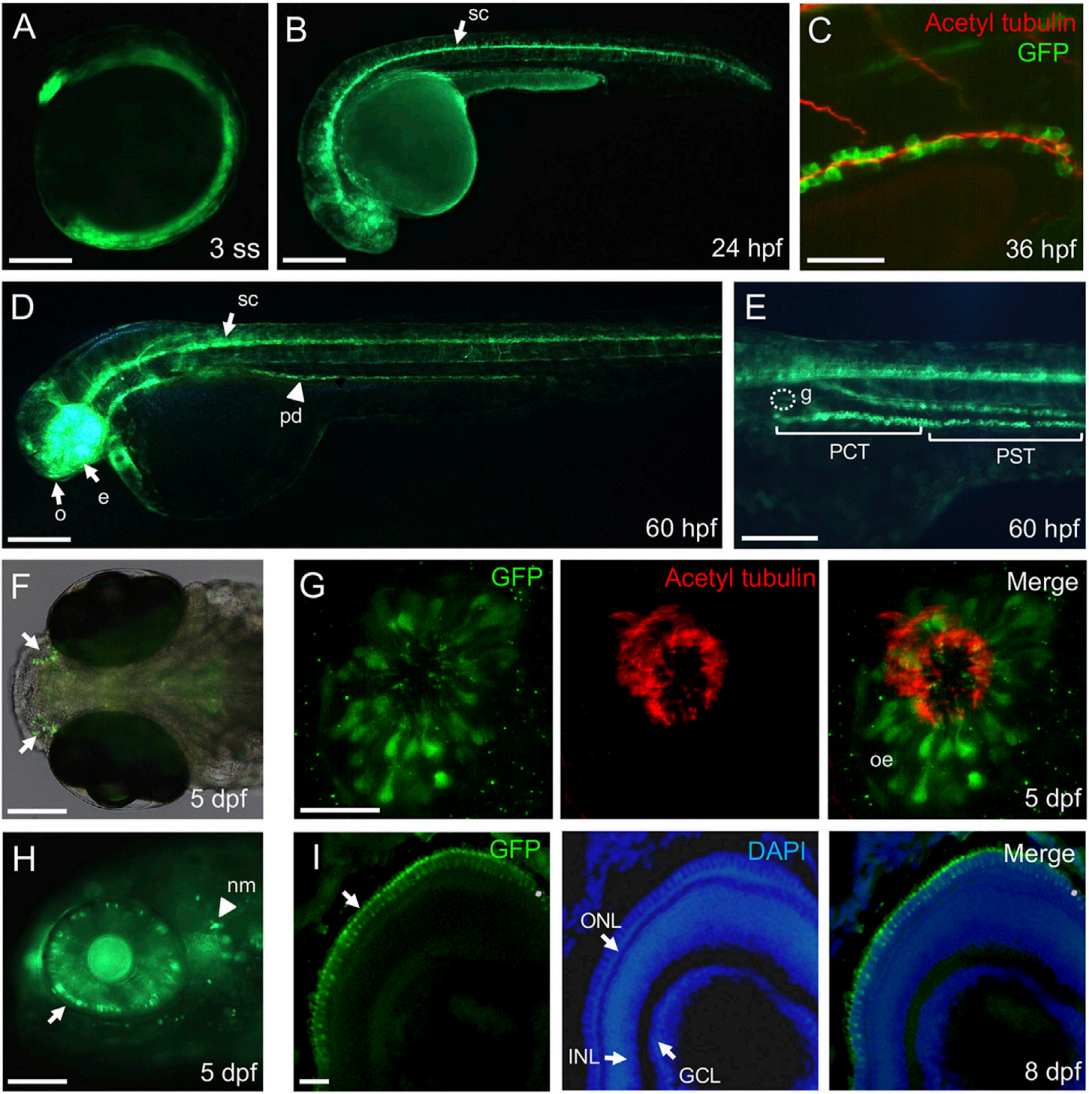
Ciliated cell-specific expression in the stable transgenic *IFT46:GAL4-VP16;UAS:mGFP* line. **(A)** Ubiquitous expression of the GFP reporter at three somite-stage embryos. **(B)** The GFP is strongly expressed in the spinal canal (arrow) at 24 hpf. **(C)** Immunostaining of anti-acetylated α-tubulin (marking cilia) with GFP in the posterior region of the pronephric duct at 36 hpf. The acetylated α-tubulin signals are overlapped on the apical region of GFP-expressing cells. **(D)** At 60 hpf, the GFP reporter is expressed in the olfactory region (o), eye (e), spinal canal (arrow), and pronephric duct (arrowhead). **(E)** High magnification image of the trunk region at 60 hpf. The GFP expression is detected in the proximal convoluted tubule (PCT) and proximal straight tubule (PST) but not in glomerulus (g). **(F)** Dorsal view of the olfactory region (arrow) in the transgenic *IFT46:GAL4-VP16;UAS:GFP* line at 5 dpf. **(G)** Immunostaining of anti-acetylated α-tubulin (marking cilia) with GFP in the olfactory region of the transgenic fish. The olfactory cilia stained with acetylated α-tubulin and GFP reporter detected in the olfactory epithelium (Oe) at 5 dpf. **(H)** The GFP expression is restricted in the outer nuclear layer of the retina and neuromast cells (nm, arrowhead) at 5 dpf. **(I)** Transverse section images of the retina at 8 dpf. The GFP reporter is expressed in photoreceptor cells in the outer nuclear layer (ONL) of the retina. Nuclei are counterstained with DAPI. INL, inner nuclear layer; GCL, ganglion cell layer. Scale bars: 200 μm **(A,B,D)**, 100 μm **(E,F,H),** 50 μm **(C)**, and 25 μm **(G,I)**.

The zebrafish pronephric duct has two types of ciliated cells, namely, multi-ciliated cells (MCCs) and single-ciliated cells (SCCs) (Vejnar et al., 2016). The clustered-motile cilia bundles formed on MCCs are observed in the anterior and middle segment of the pronephric duct, while SCCs with single-motile cilia are distributed in the posterior portion of the duct (Liu et al., 2007). We found GAL4 expression throughout the pronephros; however, GAL4 transcripts were expressed in a discontinuous pattern in the distal segment region of the pronephros at 24 hpf (Supplementary Figure S2C; Figure 1C). The MCCs are presented in the “salt and pepper” or mosaic patterns among the MCCs of the proximal convoluted tubule (PCT), proximal straight tubule (PST), and distal early (DE) tubule at 24 hpf (Wesselman et al., 2023). For more detailed examination of GAL4 expression in pronephros, we performed two-color *in situ* hybridization with *odf3b* (outer dense fiber of sperm tail 3b), a marker for MCCs, which is expressed in the distal segment of the pronephric duct (Ma and Jiang, 2007). As a result, we confirmed that *GAL4*-expressing cells overlapped with *odf3b*-expressing cells in the distal segment region (Figure 1C), indicating that *GAL4-VP16* was expressed in MCCs of the distal segment of the pronephric duct at 24 hpf. To visualize ciliated cells in live embryos, we bred *Tg(IFT46:GAL4-VP16)* to *Tg(UAS:GFP)* and *Tg(UAS:mGFP)* fish lines. GFP expression was first detectable at early somite-stage embryos (Figure 2A). Like *GAL4* expression, the GFP reporter was expressed in the eye, spinal canal, and pronephric duct at 24 hpf (Figure 2B). To evaluate whether GFP-expressing cells are ciliated in pronephric ducts, we performed immunostaining with the anti-acetylated α-tubulin antibody at 36 hpf. GFP-expressing cells were positive for anti-acetylated α-tubulin in pronephric ducts (Figure 2C). Transverse sections of *Tg(IFT46:GAL4-VP16;UAS:GFP)* indicated that GFP-positive cells are a subset of epithelial cells in pronephric ducts at 48 hpf (Supplementary Figure S2G). We also found GFP expression in the olfactory pit, eye, and spinal canal of the central nervous system (CNS) as well as in the PCT of the pronephros at 60 hpf (Figures 2D, E) (Wingert and Davidson, 2008; Drummond and Davidson, 2016). In olfactory regions, the GFP reporter was bilaterally expressed in the olfactory epithelium that was co-stained with acetylate α-tubulin at 5 dpf (Figures 2F, G). At 5 dpf, GFP expression was found in the outer nuclear cell layer (ONL) of the retina and neuromast cells (Figure 2H). In addition, GFP reporter expression was restricted to photoreceptor cells in the ONL of the retina at 8 dpf (Figure 2I). Taken together, these results indicate that the *IFT46:GAL4-VP16* transgenic line can directly control GFP expression in various ciliated tissues during early embryogenesis.

### 3.3 Nitroreductase-mediated ciliated cell ablation using the Tg(*IFT46:GAL4-VP16*; UAS:GFP) line

**FIGURE 3:**
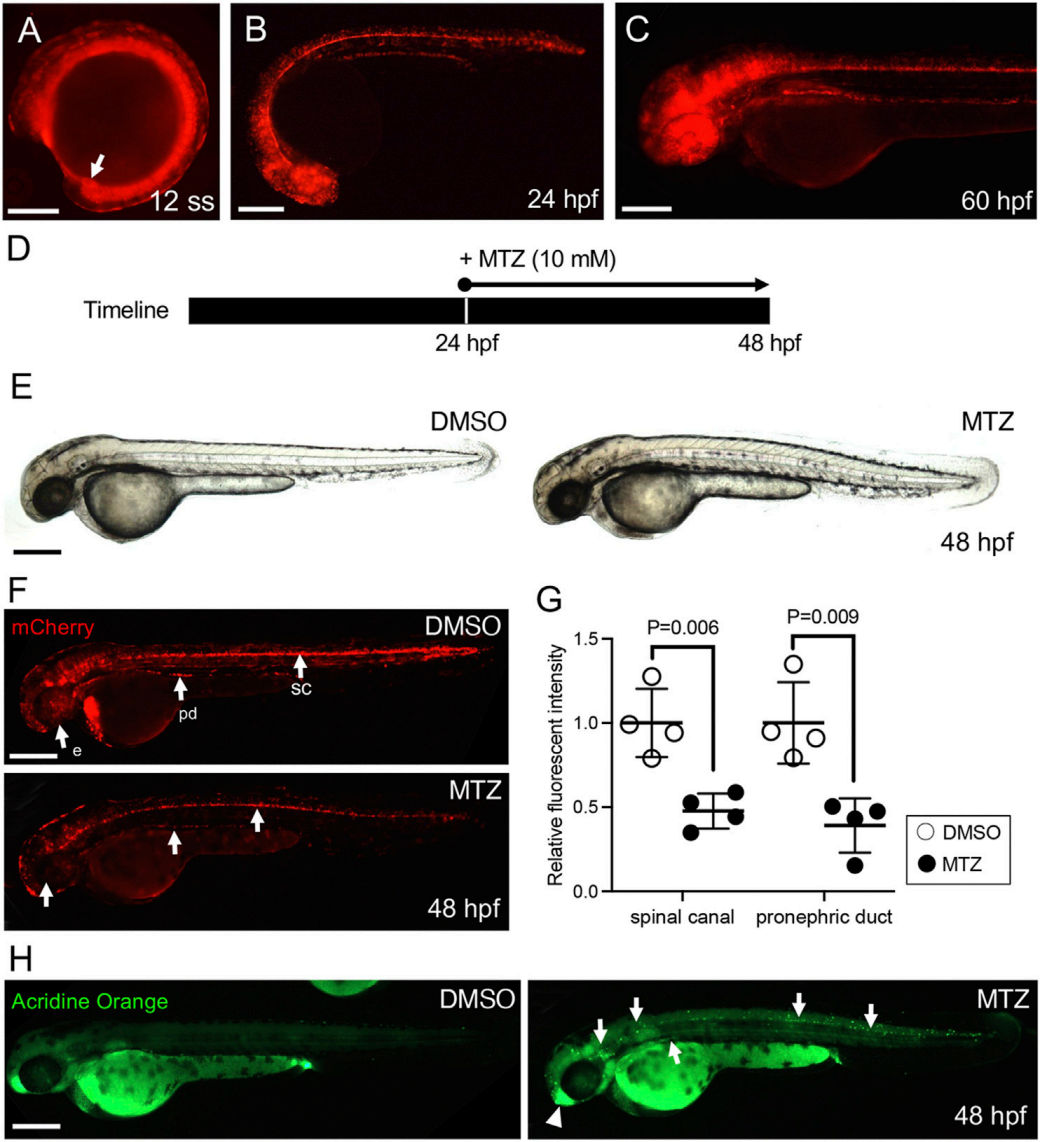
Establishment of the ciliated cell-specific ablation zebrafish model. **(A)** The mCherry reporter expression in a stable transgenic *IFT46:GAL4-VP16;UAS:nsfb-mCherry* line at the 12 somite stage. The arrow indicates Kupffer’s vesicle. **(B,C)** mCherry is expressed in ciliated organs including the eye, olfactory region, spinal canal, and pronephric duct at 24 hpf **(B)** and 60 hpf **(C)**. Scale bar = 200 μm. **(D)** Schematic timeline of MTZ treatment in transgenic embryos. **(E)** Gross morphology of MTZ-treated 48 hpf zebrafish embryos. **(F)** The mCherry expression in MTZ-treated 48 hpf larvae. The MTZ-treated embryos show decreased mCherry signals in the spinal canal, pronephric duct, and eye. **(G)** The fluorescence intensity of mCherry signals is significantly decreased in both the spinal cord and pronephric duct in MTZ-treated embryos. Error bars are the mean ± S.E.M; *p*-values are determined by the unpaired Mann–Whitney test (*p = 0.006 and *p = 0.009). **(H)** The MTZ-treated embryo showed increased acridine orange-positive cell death in the spinal canal, pronephric duct, and olfactory region in mCherry-expressing cells. Scale bars: 200 μm **(A,B,C,E,F,H)**.

To investigate the role of ciliated cells during organ development, the *Tg*(*IFT46:GAL4-VP16*) line was crossed with *Tg*(*UAS:nfsb-mCherry*) to induce ciliated cell death using the NTR/MTZ cell ablation system (Curado et al., 2007; Pisharath et al., 2007; White and Mumm, 2013; Gregoire and Kmita, 2014). Similar to our observations in *Tg*(*IFT46:GAL4-VP16;UAS:GFP*), mCherry expression was detected in Kupffer’s vesicle at the mid-somite stage and restricted to ciliated tissues after 24 hpf (Figures 3A–C). To test whether the NTR/MTZ-based cell ablation system works properly in our transgenic line, we treated embryos with 10 mM MTZ from 24 hpf to 48 hpf and observed for morphological changes (Figure 3D). DMSO- and MTZ-treated embryos did not exhibit any significant morphological differences at 48 hpf (Figure 3E). However, mCherry expression in the eye, spinal canal, and pronephric duct was dramatically reduced in MTZ-treated embryos compared to DMSO-treated control (Figures 3F, G). To assess whether the reduction in mCherry expression in MTZ-treated transgenic embryos is due to cell death in ciliated cells by NTR cytotoxicity, we performed acridine orange staining, which can detect cell death *in vivo*, and found an increased number of dying cells in MTZ-treated embryos (Figure 3H). Most dying cells were mCherry-expressing cells in MTZ-treated embryos (Supplementary Figure S3A). These results clearly suggest that NTR/MTZ-based cell ablation can be utilized to perform inducible zebrafish-ciliated cell ablation.

### 3.4 Conditionally ablated ciliated cells cause ciliopathy-like phenotypes

**FIGURE 4:**
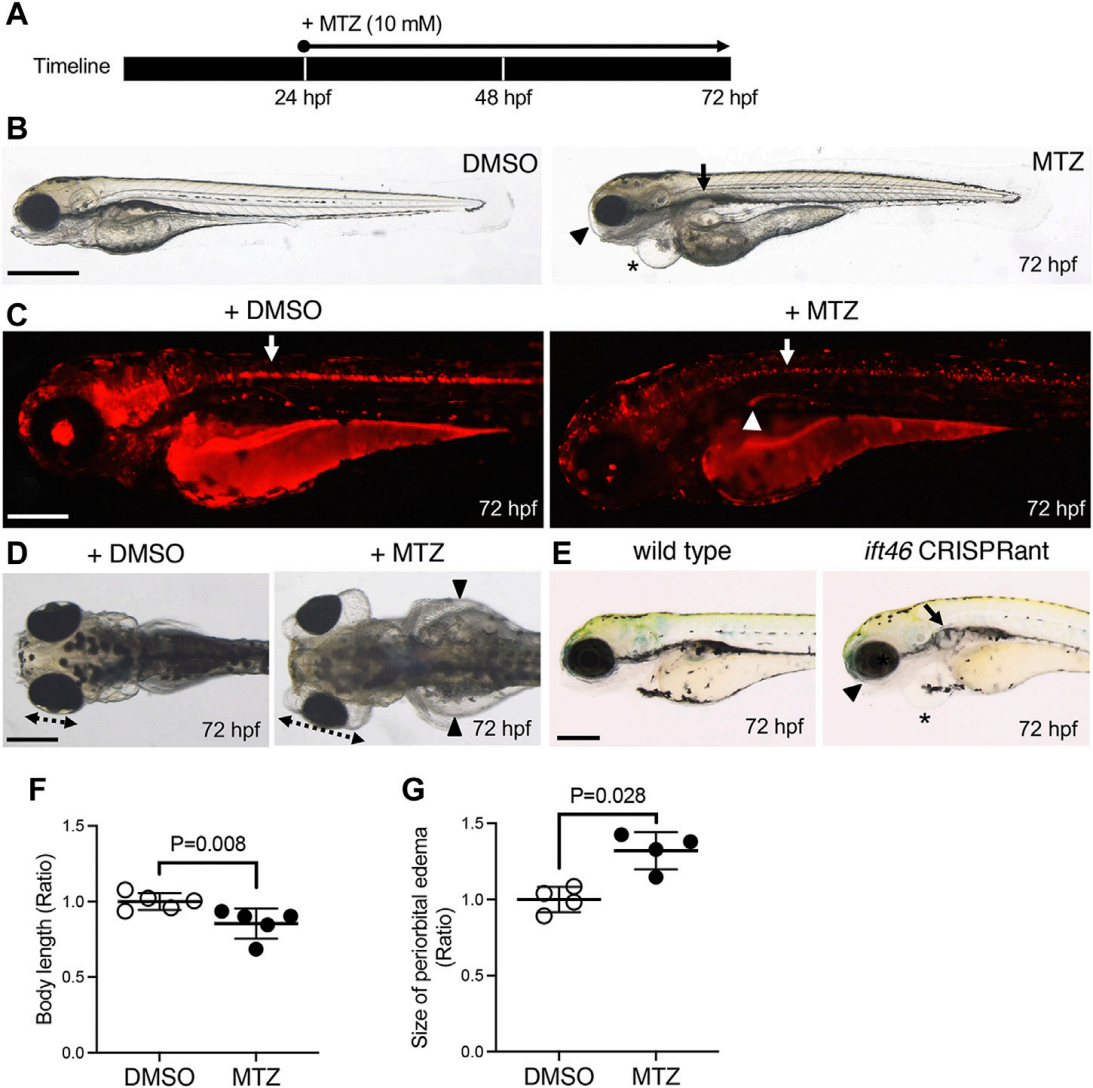
Ciliopathy zebrafish model by ciliated cell ablation in *Tg*(*IFT46:GAL4-VP16;UAS:nsfb-mCherry*). **(A)** Schematic timeline of MTZ treatment in transgenic embryos. **(B)** The MTZ-treated embryos exhibit shortened body length, eye edema (arrowhead), cardiac edema (asterisk), and cystic kidney (arrow) at 72 hpf. **(C)** mCherry signals are significantly decreased in the spinal canal (arrow) and pronephric duct (arrowhead) after MTZ-treated larvae compared to DMSO-treated larvae. **(D)** Gross morphology in the anterior region of DMSO and MTZ-treated larvae at 72 hpf. The MTZ-treated larvae show severe whole-body edema (arrowheads), cystic kidney (arrow), and periorbital edema. The dotted lines indicate the eye size. **(E)** Morphological phenotypes of *IFT46* CRISPRant at 72 hpf. CRISPRant exhibits periorbital edema (arrowhead), cardiac edema (asterisk), and cystic kidney (arrow). **(F,G)** Quantification of whole-body length (based on the length of head to end of tail fin) **(F)** and periorbital edema size **(G)** in MTZ-treated larvae compared to DMSO-treated larvae at 72 hpf. Error bars are the mean ± S.E.M; *p*-values are determined by the unpaired Mann–Whitney *U*-test (*p = 0.008 and *p = 0.028). Scale bars: 500 μm **(B)** and 250 μm **(C–E)**.

Like our previous report, most genetic mutations and/or knockdown of ciliogenesis-related genes exhibit ciliopathy-like phenotypes including body axis curvature, cystic kidneys, and defective left–right patterning caused by ciliary dysfunction or defects (Kramer-Zucker et al., 2005; Houde et al., 2006; Zhao and Malicki, 2007; Delaval et al., 2011; Lee et al., 2015). To establish a cilium-injury model, we conditionally ablated ciliated cells in Tg (*IFT46-NTR-mCherry*) with MTZ. First, we treated MTZ to 24 hpf transgenic embryos for 48 h (Figure 4A). At 72 hpf, MTZ-treated transgenic larvae exhibited shortened body size, a darkened yolk sac, and head region as well as decreased mCherry expression, indicating that NTR-expressing ciliated cells underwent cell death (Figures 4B, C). Notably, a decreased number of *odf3b* (marking MCCs)-positive cells were observed at 48 hpf and 72 hpf MTZ-treated transgenic larvae (Supplementary Figures S3A, B). In addition, MTZ-treated transgenic larvae showed ciliopathy-like phenotypes including severe periorbital and pericardial edema as well as cystic kidneys compared to DMSO-treated transgenic larvae (Figure 4D). We found that the whole-body length was decreased, while periorbital edematous size was increased in MTZ-treated transgenic larvae compared to DMSO-treated larvae (Figures 4F, G) (Zhou and Hildebrandt, 2012; Wan et al., 2016; Lee et al., 2022). As concern from morpholino-mediated knockdown may give non-specific phenotypes, we generated *IFT46* F0 zebrafish using the CRISPR/Cas9 system (Supplementary Figure S4A). We microinjected three gRNAs along with Cas9 mRNA into the one-cell stage embryos and observed mutations in the target site by using the T7 endonuclease I assay (Supplementary Figure S4A). The *IFT46* CRISPRants exhibit ventral body curvature, pericardiac and periorbital edema, and cystic kidneys (27%, *n* = 30/100) at 3 dpf (Figure 4E). Similar to MTZ-treated transgenic larvae, the edematous phenotypes of the CRISPRants were progressively severe and eventually led to whole-body edema (Supplementary Figure S4C).

To control NTR/MTZ-induced ciliated cell death temporally, we incubated 36 hpf transgenic embryos with MTZ for 12 h and confirmed reduced mCherry expression where the *IFT46* promoter was activated. NTR-induced cell death was observed in the spinal canal and pronephric duct at 48 hpf (Supplementary Figures S5A, B). This result indicates that the incubation of MTZ for 12 h is enough to induce ciliated cell ablation in the transgenic line. Additionally, we treated MTZ to 36 hpf transgenic embryos for 24 h and found that larvae exhibited mild pericardial edema (Supplementary Figure S5C). Furthermore, when 36 hpf transgenic embryos were treated with MTZ for 3 days, the larvae exhibited pericardial edema, cystic kidneys, and mild body curvature (Supplementary Figure S5E). These results indicated that the NTR-mediated ciliated cell injury model can consistently induce ciliopathy-like phenotypes including small eye, pericardial edema, and cystic kidney at different times.

## 4 Discussion

In the present study, we introduced an inducible ciliopathy model in zebrafish by cilium cell-specific ablation using the NTR/MTZ system. The advantages of using zebrafish as a model organism for human ciliopathies are the variety of specialized cilia types present in developing embryos, like in humans, as well as the possibility of live imaging and characterization during early development (Zhao and Malicki, 2007). In addition to the high conservation of cilia-related genes and cilia types between zebrafish and humans, the strong morphological and functional conservation of cilia in various organs validate the use of the zebrafish model system to study human ciliopathies (Song et al., 2016). Moreover, genetic mutations in cilium-related genes display typical ciliopathy phenotypes such as cystic kidneys or retinal dystrophy and degeneration as well as body axis curvature (Houde et al., 2006; Zhao and Malicki, 2007; Delaval et al., 2011). As with these phenotypic changes, it is possible to identify and isolate genetic mutants affecting cilium formation and function. Similar to phenotypes in genetic mutants or morphants of cilium-related genes, our transgenic animals exhibited ciliopathy-like phenotypes after cilium cell-specific ablation. Thus, zebrafish can be used as a ciliopathy model in the study of human ciliopathies.

IFT proteins play an essential role in cilium growth and structure. IFT-B mutations often cause defects in ciliogenesis, whereas the absence of IFT-B can induce an abnormal accumulation of other IFT proteins in cilium tip (Shi et al., 2018). We previously reported that *IFT46* is expressed in ciliated organs during zebrafish early development and that knockdown of *IFT46* exhibited ciliopathy-related phenotypes (Lee et al., 2015). Here, we extended our findings to characterize the upstream regulatory elements of the *IFT46* gene, which can control the expression of effector molecules including fluorescent reporters or reductase in ciliated organs. The 2.4 kb upstream regulatory sequences of the *IFT46* promoter were sufficient to drive the expression of the fluorescent reporters in various ciliated tissues during embryonic development. Moreover, we used the GAL4-VP16 chimeric transcriptional activator as a driver for temporal expression of effector molecules in ciliated organs. However, ectopic expression of reporters was observed in skeletal muscle (Supplementary Figures S2C, D). This might be due to the strong expression of the GAL4-VP16 activator or because the 2.4 kb upstream regulatory sequence was not perfectly suitable to recapitulate the endogenous expression of the *IFT46* gene.

NTR/MTZ-mediated cell ablation has become a popular method in animal models, where it has expanded studies of cellular function and tissue regeneration in vertebrate systems. The key advantage of MTZ is the cell-specific nature of its cytotoxic metabolites, allowing selective ablation of NTR-expressing cells without the side effects to surrounding cells. The targeted ablation enables precise elimination of cellular subtypes and subsequent analysis of their roles during developmental, regenerative, and other biological processes of interest (Curado et al., 2007; Curado et al., 2008; White and Mumm, 2013; Gregoire and Kmita, 2014). Here, we focused on 2–5-day zebrafish larvae for inducing ciliopathy phenotypes with 10 mM MTZ as a standard protocol. Many cases of mutants and morphants exhibiting ciliary defects have demonstrated phenotypes such as body curvature, cystic kidney, and periorbital edema at these developmental stages (Houde et al., 2006; Zhao and Malicki, 2007; Delaval et al., 2011). Indeed, we established a zebrafish ciliopathy model as a phenotypic consequence by cilium cell-specific injury. Additionally, we have shown the utility of the NTR/MTZ system to induce ciliopathy phenotypes by specifically restricting cell death-inducing effects of NTR to ciliated cells. As the system is temporally inducible, highly efficient, and the duration of the ablation can be controlled by incubation time, it would be possible to study late onset ciliopathy based on different concentration and incubation time of MTZ on adult transgenic animals.

In conclusion, we introduced the advantages of the *Tg(IFT46:GAL4-VP16;UAS:nfsb-mCherry)* line, which enables observation of ciliated cells and modeling for human ciliopathy. Additionally, our transgenic animals can serve as a versatile and dependable model for *in vivo* monitoring of ciliopathy controlled by cilium cell-specific injury. This ciliopathy animal model might have advantages in further investigations for identifying signaling pathways or new molecules involved in the process of cilium cell regeneration according to tissue—retinal dystrophy in the eyes, anosmia of the nose, congenital heart defects, renal anomalies of the kidneys, etc.

## Data Availability

The original contributions presented in the study are included in the article/Supplementary Material; further inquiries can be directed to the corresponding authors.

## Abbreviations

IFT, intraflagellar transport; NTR, nitroreductase; MTZ, metronidazole; PAM, protospacer-adjacent motif; PKD, polycystic kidney disease; PCD, primary ciliary dyskinesia; RFX, regulatory factor X; CNS, central nervous system; MCCs, multi-ciliated cells; SCCs, single-ciliated cells; PCT, proximal convoluted tubule; PST, proximal straight tubule; DE, distal early; Pd, pronephric duct; Sc, spinal canal; Oe, olfactory epithelium; Op, olfactory pit; Mn, neuromast; Pl, photoreceptor cell layer; ONL, outer nuclear layer; INL, inner nuclear layer; GC, ganglion cell layer; PCT, proximal convoluted tubule; T7E1, T7 endonuclease I; UAS, upstream activation sequence.
