# Supplementary material for: IFT46 gene promoter-driven ciliopathy disease model in zebrafish
Source: Front Cell Dev Biol. 2023 Jun 8;11:1200599. doi: 10.3389/fcell.2023.1200599 (PMC10285392; doi:10.3389/fcell.2023.1200599)
Supplement: Supplementary file 1 [file DataSheet1.docx]

**Supplementary Materials**

**Supplementary Figures**

**
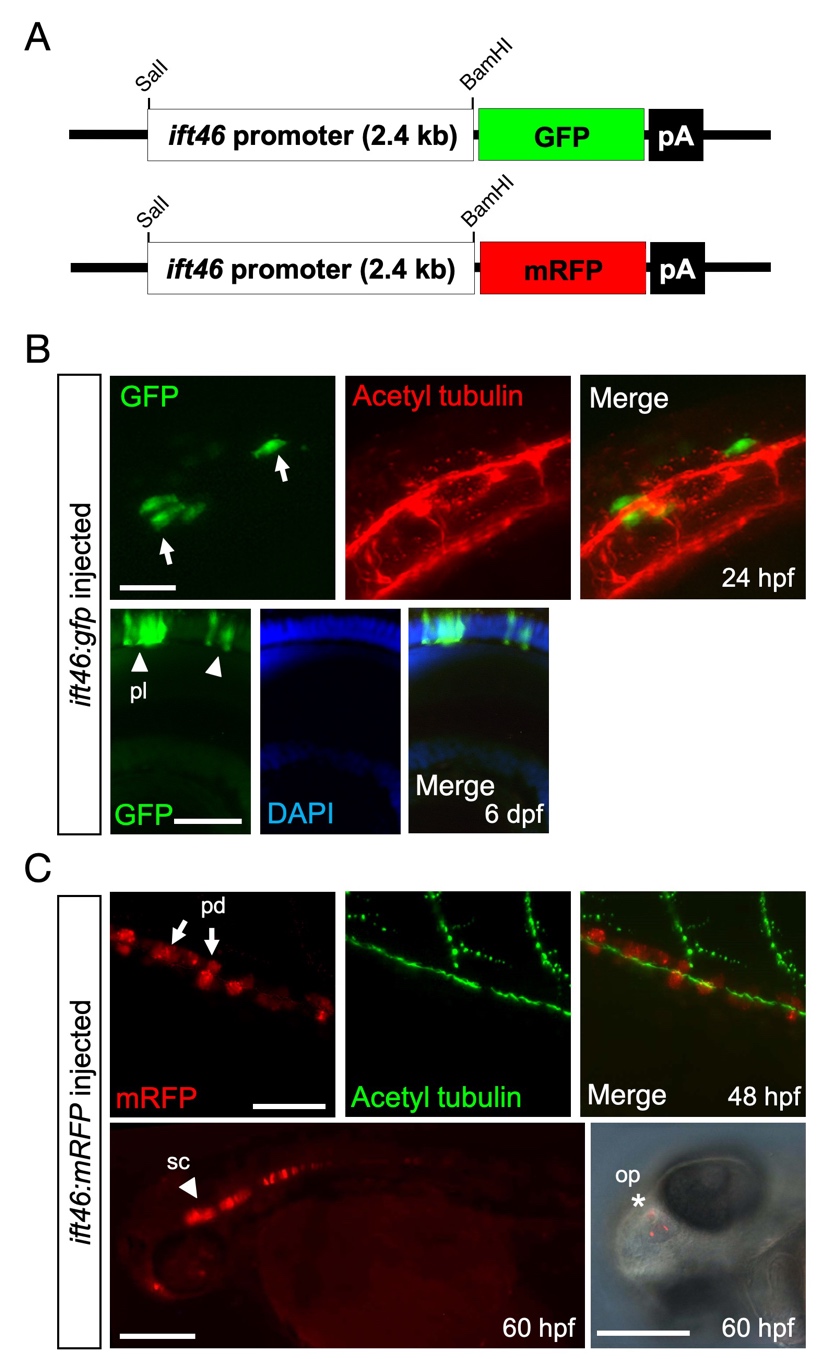
**

**Figure S1. Transient expression of reporters under control of 2.4 kb *ift46* promoter.** (A) Schematic constructs of *ift46:GFP* and *ift46:mRFP*  (B) Transient expression of *ift46:GFP* construct during early development. Mosaic expression of GFP reporter is observed in ear (arrows) at 24 hpf and photoreceptor cells (arrowheads) at 6 dpf. (C) The mRFP reporter is detected in pronephric duct (arrows) at 48 hpf and spinal canal (arrowhead) and olfactory (asterisk) at 60 hpf. Scale bars: 50 μm (B, C).

**
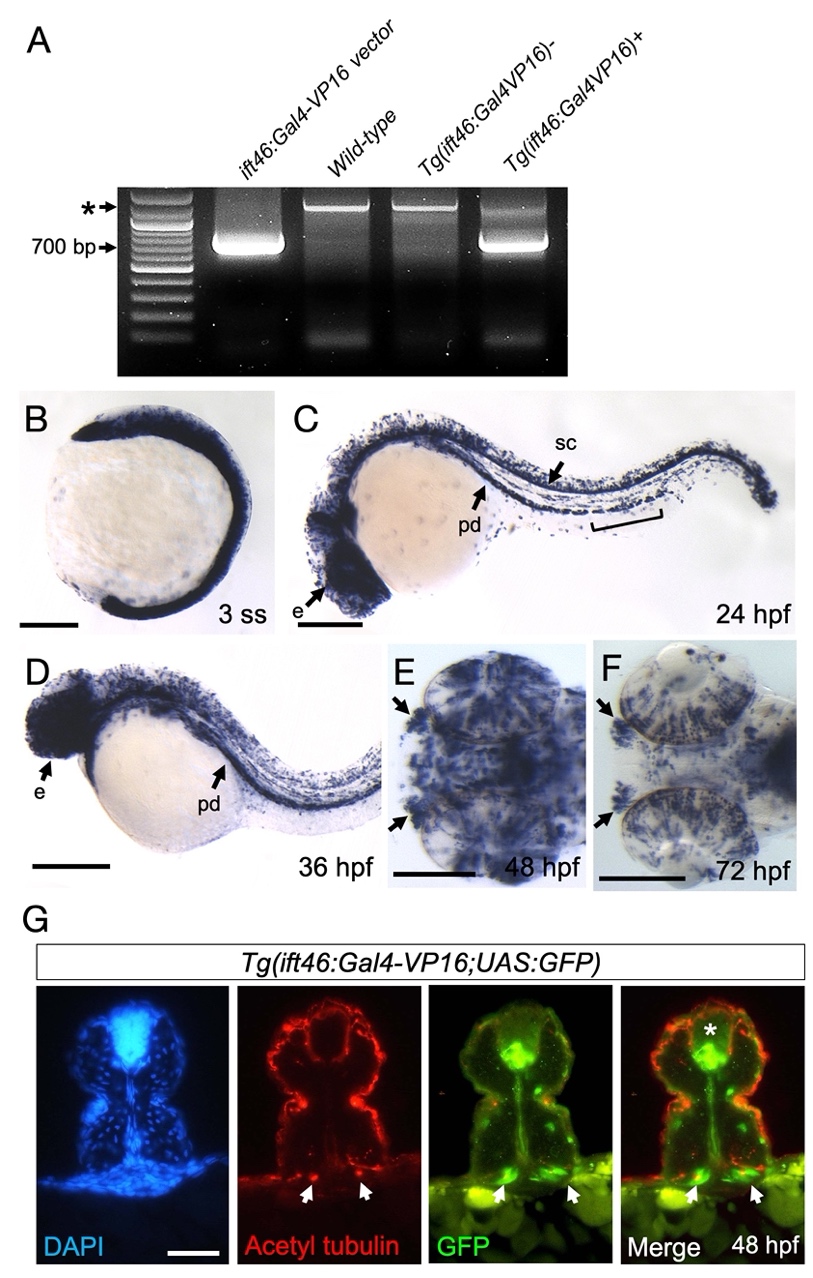
**

**Figure S2. Establishment of a stable transgenic *Tg(ift46:Gal4-VP16)* line.** (A) *ift46:Gal4-VP16* transgenic is determined by genomic PCR from 2-month-old F1 fish fin clips using Gal4 gene specific primer. *mini-Tol2-ift46:Gal4-VP16* vector used for positive control. The expected size of the *Gal4* amplification is 700 bp. Asterisk indicates non-specific bands. (B-F) Expression pattern of *Gal4-VP16* transcripts by whole-mount *in* *situ* hybridization during early development. (B) *Gal4-VP16* transcripts are expressed ubiquitously at 3 somite stage. At 24 hpf (C) to 36 hpf (D), the transcripts are detected in various ciliated tissues including eye (e), spinal canal (sc) and pronephric duct (pd) (arrows). The black line indicates distal segment of pronephros (C). (E, F) Dorsal views for *Gal4* expressing olfactory region (arrows) and retina at 48 hpf (E) and 72 hpf (F). (G) Transverse section image of trunk region at 48 hpf. GFP expression of *Tg(ift46:Gal4-VP16;UAS:GFP)* line is observed in epithelial cells of pronephric duct (arrows) and spinal canal (asterisk). Anti-acetylated α-tubulin (marking cilia) signals are co-localized with GFP signals in pronephric duct and spinal canal. Scale bars: 200 μm (B, C, D), 100 μm (E, F), and 50 μm (G).


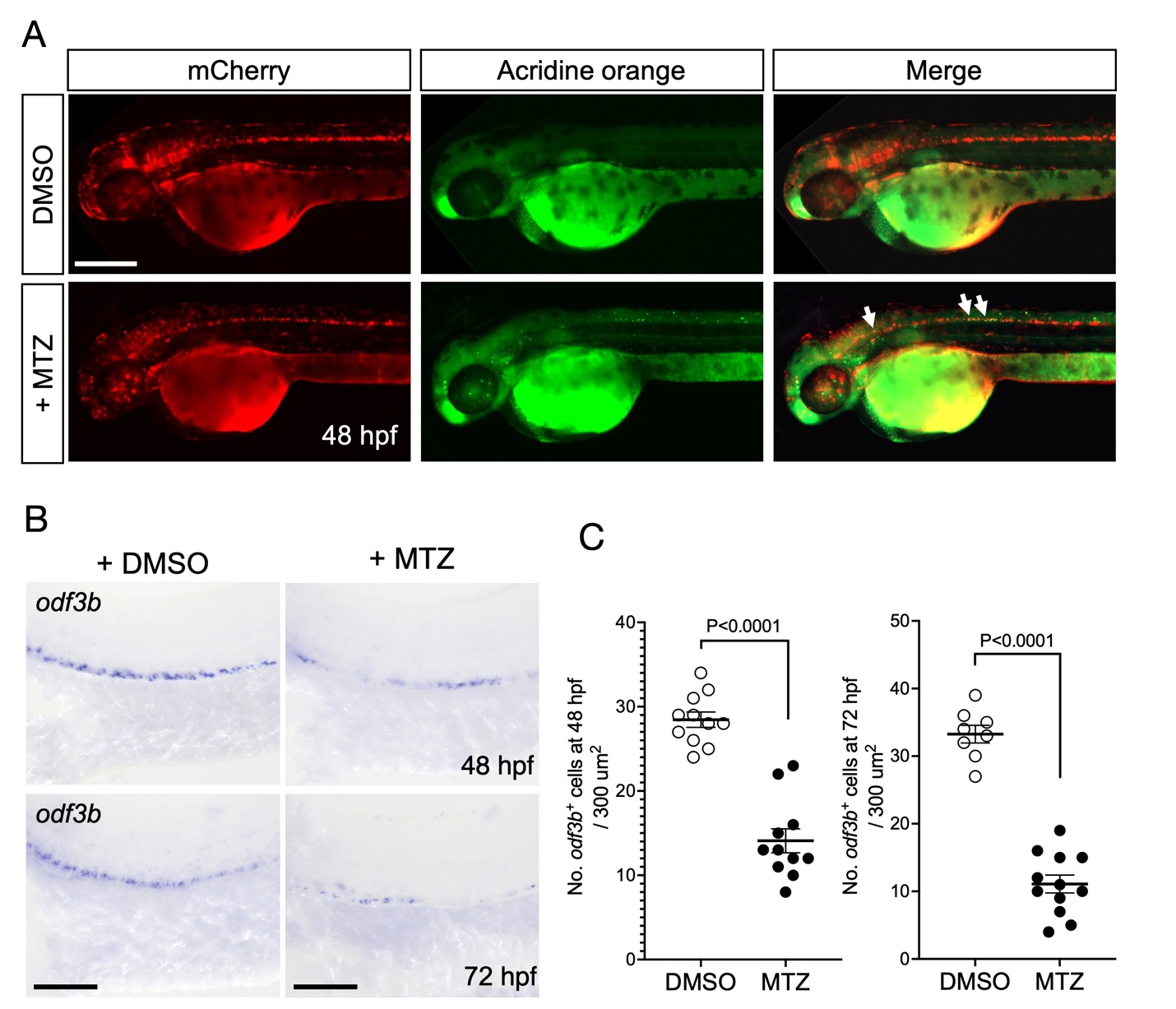


**Figure S3. Ciliated cell-specific ablation by MTZ treatment in *Tg(ift46:Gal4-VP16;UAS:nsfb-mcherry)* line.** (A) At 48 hpf, acridine orange-positive dying cells are specifically detected in spinal canal (arrows) where mCherry-expressing cells locate. (B) Whole-mount *in situ* hybridization for odf3b (marking MCCs) in MTZ-treated 48 hpf and 72 hpf transgenic larva. (C) Quantification of *odf3b*-positive cell number in MTZ-treated 48 hpf and 72 hpf transgenic larva. Error bars are means ± S.E.M; *p*-values are determined by unpaired Mann-Whitney test. Scale bars: 250 μm (A) and 100 μm (B).


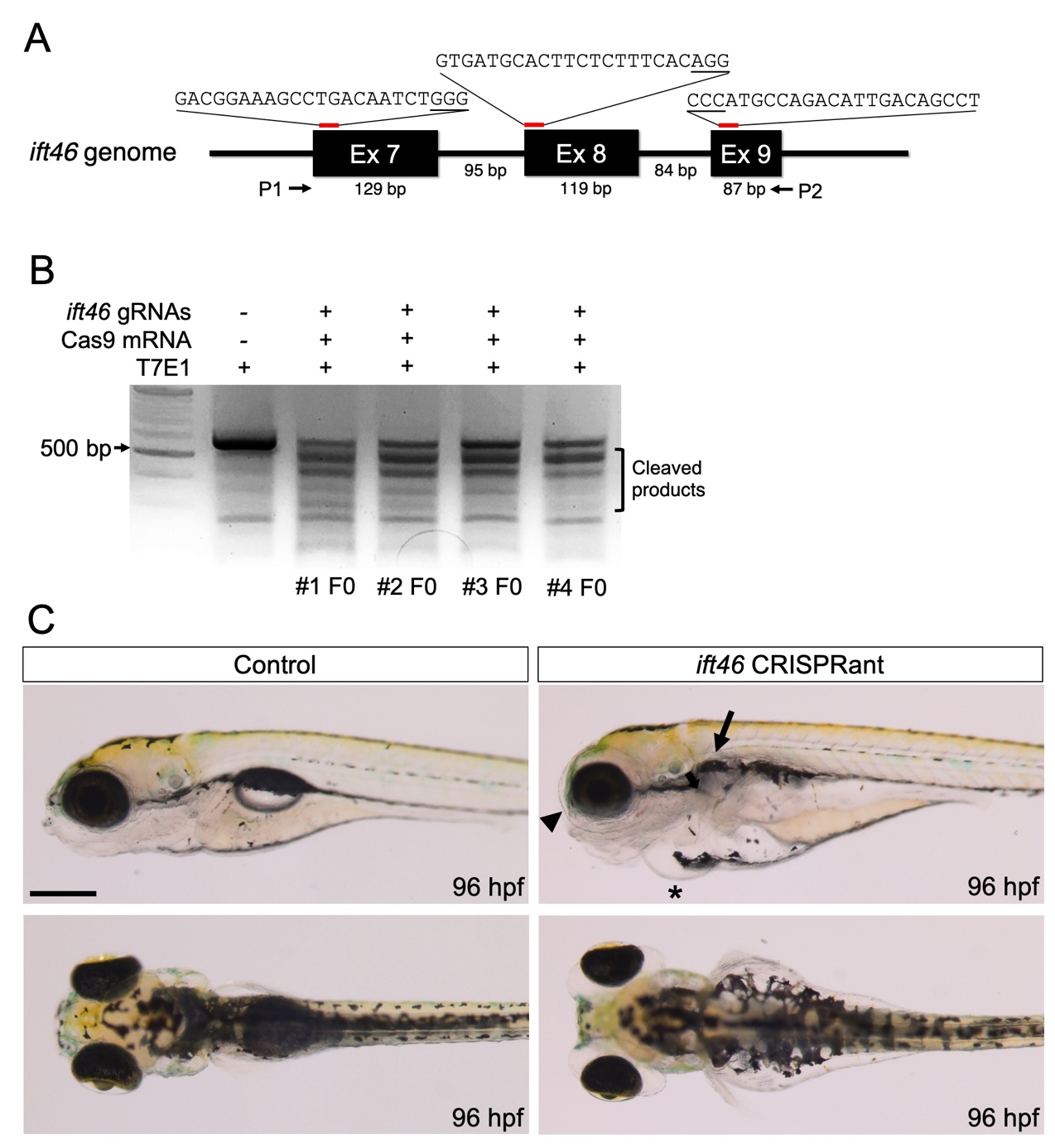


**Figure S4. CRISPR/Cas9-mediated *ift46* gene editing** (A) The target sequence of guide RNAs (gRNAs) on *ift46* genome. The three gRNAs were designed to target on exon 7, 8, and 9. Protospacer-adjacent motif (PAM) sequences are underlined. (B) CRISPR/Cas9-induced *ift46* mutations detected by T7 endonuclease I (T7E1) assay at 72 hpf F0 founder larva . The size of PCR amplification from P1 (forward primer) and P2 (reverse primer) is 550 bp. Bracket points to cleaved products, indicating T7E1 nuclease activity. (C) Gross morphology of uninjected control and *ift46* CRISPRant at 96 hpf. The *ift46* CRISPRant exhibits pericardiac edema (asterisk), periorbital edema (arrowhead) with small retina, and cystic kidney (arrow) at 96 hpf. Scale bar: 250 μm.

**
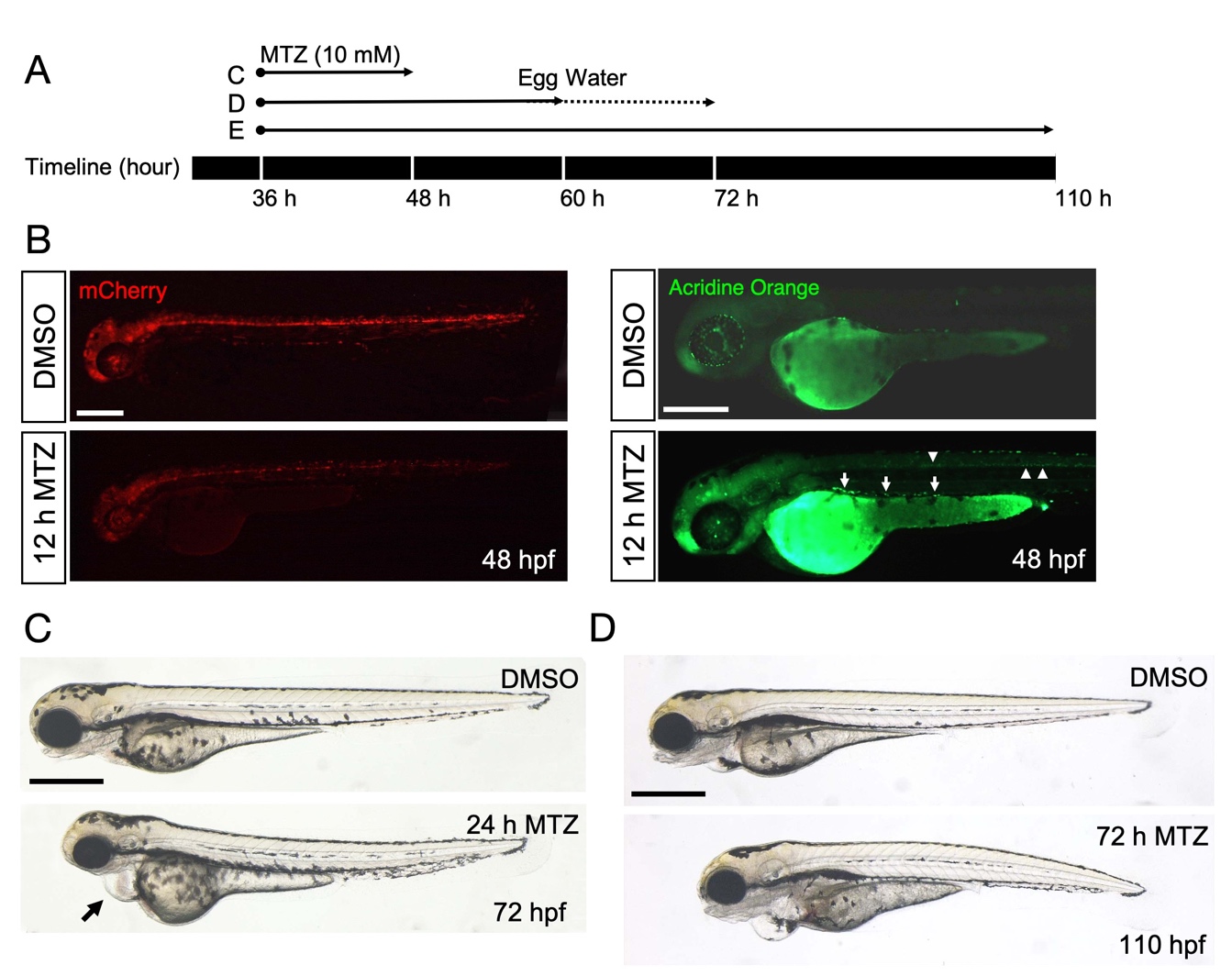
**

**Figure S5. Long-term effects of ciliated cell-specific ablation by MTZ treatment in *Tg(ift46:Gal4-VP16;UAS:nsfb-mcherry)* line.** (A) Schematic timeline for MTZ treatment in transgenic embryos. (B) At 48 hpf transgenic embryos treated with MTZ for 12 hours at 36 hpf, the mCherry expression is significantly decreased in ciliated organs. Acridine orange-positive dying cells are detected in spinal canal (arrowheads) and pronephric duct (arrows). (C) At 72 hpf transgenic larvae treated with MTZ for 24 hours and washed with egg water for 12 hours, the larvae exhibit cardiac edema (arrow). (D) Transgenic larvae treated with MTZ for 3 days exhibit ciliopathy-like phenotypes. Scale bars: 500 μm (C, D) and 250 μm (B).
